# Abnormal X : autosome ratio, but normal X chromosome inactivation in human triploid cultures

**DOI:** 10.1186/1471-2156-7-41

**Published:** 2006-07-03

**Authors:** Stanley M Gartler, Kartik R Varadarajan, Ping Luo, Thomas H Norwood, Theresa K Canfield, R Scott Hansen

**Affiliations:** 1 Department of Medicine, Division of Medical Genetics,, University of Washington, Seattle, WA 98195, USA; 2 Department of Genome Sciences, University of Washington, Seattle, WA 98195, USA; 3 Department of Pathology, University of Washington, Seattle, WA 98195, USA

## Abstract

**Background:**

X chromosome inactivation (XCI) is that aspect of mammalian dosage compensation that brings about equivalence of X-linked gene expression between females and males by inactivating one of the two X chromosomes (Xi) in normal female cells, leaving them with a single active X (Xa) as in male cells. In cells with more than two X's, but a diploid autosomal complement, all X's but one, Xa, are inactivated. This phenomenon is commonly thought to suggest 1) that normal development requires a ratio of one Xa per diploid autosomal set, and 2) that an early event in XCI is the marking of one X to be active, with remaining X's becoming inactivated by default.

**Results:**

Triploids provide a test of these ideas because the ratio of one Xa per diploid autosomal set cannot be achieved, yet this abnormal ratio should not necessarily affect the one-Xa choice mechanism for XCI. Previous studies of XCI patterns in murine triploids support the single-Xa model, but human triploids mostly have two-Xa cells, whether they are XXX or XXY. The XCI patterns we observe in fibroblast cultures from different XXX human triploids suggest that the two-Xa pattern of XCI is selected for, and may have resulted from rare segregation errors or Xi reactivation.

**Conclusion:**

The initial X inactivation pattern in human triploids, therefore, is likely to resemble the pattern that predominates in murine triploids, i.e., a single Xa, with the remaining X's inactive. Furthermore, our studies of XIST RNA accumulation and promoter methylation suggest that the basic features of XCI are normal in triploids despite the abnormal X:autosome ratio.

## Background

Dosage compensation in mammals is normally achieved by the transcriptional silencing of one of the two X chromosomes (Xi) in females early in development (X chromosome inactivation; XCI). It is widely assumed that an early event in XCI is a counting mechanism that senses the X chromosome:autosome ratio, such that one X chromosome per diploid autosomal set remains active, with additional X's rendered inactive. [1-3]. In triploids, which occur in approximately 1% of human conceptions [4], this ratio of one active X (Xa) per diploid autosomal set cannot be achieved. If developmental steps are associated with checkpoints, it is likely that development in triploids would cease at this point. This is not the case, however, as development in triploids proceeds well beyond the X inactivation window, with some cases reaching term. A variety of XCI patterns have been observed in triploids; in XXX triploids, for example, single inactive X cells (XaXaXi) and double inactive X cells (XaXiXi) have been found in independent cultures as well as in the same (mosaic) cultures [5]. Jacobs et al. [5] proposed that the X:autosome ratio in triploids might cause XCI to be unstable, resulting in cells that shift between one and two Xi's in XXX's, and one and no Xi's in XXY's. A limited set of data from a study of clones from a human XXY triploid culture [6], and more extensive clonal studies on human XXX triploids that we report here, argue against this possibility.

A number of studies analyzed sex chromatin to determine the number of Xi's in human triploid fetal cells [7], and a single major study analyzed late replicating X's for the same purpose [5]. The conclusions from these studies are similar: for XXY triploids the majority of cells at the stages studied are XaXaY, and for XXX triploids, the majority of cells are XaXaXi. In contrast to fetal cells, cells from live-born triploids, of which there are few, appear to show a higher proportion of Xi's: single Xi's in XXY's, and two Xi's in XXX's [7]. The origin of human triploidy could have some bearing on this question. The majority of human triploids are diandric in origin and usually result in spontaneous abortions, whereas the majority of late surviving triploids, of which there are few, appear to be largely of digynic origin [4]. The only extensive studies in an experimental animal were in the mouse, where the results are quite clear for embryos: XXX triploids are mainly XaXiXi, and XXY's are mainly XaXiY [8,9].

Our work on human XXX triploid fibroblasts revealed that most of the cultures consisted primarily of cells with one Xi, in agreement with earlier studies on human fetal specimens. Early passage cultures from two different newborn triploids each contained relatively high levels of cells with two Xi's. This pattern changed in later passages to predominantly single-Xi cells, suggesting that selection for cells with two Xa's occurs, at least during normal culture. Clonal analyses of the two- and one-Xi cell classes showed that they bred true, indicating that the X inactivation state is stably maintained in triploid cells. We also found the fluorescent *in situ *hybridization (FISH) signals for XIST RNA in triploids to be similar in intensity and morphology to those in normal female cells, arguing against the possibility that XCI could be abnormal in triploids because of a quantitative change in functional XIST RNA levels. Finally, we examined DNA methylation patterns at the X-linked *G6PD *locus in the various triploid cultures, and found that the ratio of hypomethylated to hypermethylated alleles agreed well with the expected ratios of Xa:Xi determined by FISH analyses of XIST RNA and X chromosome DNA.

## Results and discussion

### XIST RNA bodies

**Figure 1:**
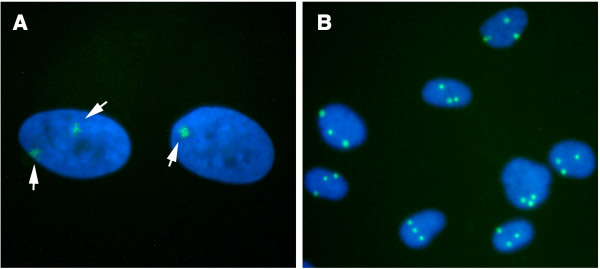
Counting Xi's and X's in control and triploid fibroblasts. A. XIST RNA FISH was used to determine Xi content in cells. In this example, two cells from an early passage of 75-29 XXX triploid fibroblasts are shown, one with a single XIST RNA body, and the other with two (arrows). B. X chromosome content was determined using FISH to X-linked alpha centromeric DNA (CEP X probe). In this example, cells from a clone of 75-29 (F10) all show three X-centromere signals.

To examine X inactivation status in triploid cultures, we first performed XIST RNA FISH analysis on six XXX triploid cultures at early passage, and three control cultures (normal female, normal male, and a trisomy X culture). Figure 1A provides an example of this type of analysis. Over 95% of the cells could be confidently scored, and the control cells exhibited the expected patterns of XIST RNA signals (Table 1). All six triploids were mosaic, with some cells having single XIST RNA bodies (XaXaXi), and others having two (XaXiXi) (Table 1; Fig. 1A). Four of the triploid cultures were mainly XaXaXi, varying from 90% to over 99% XaXaXi. Two of the triploid cultures, GM04939 and 75-29, were quite different in this respect: GM04939 had approximately equal numbers of XaXaXi and XaXiXi cells, while 75-29 had approximately 70% XaXiXi and 30% XaXaXi cells.

**Table 1:** XIST RNA signals and X chromosome counts in control and XXX triploid cultures

**Culture**^a^	**Karyotype**	**XIST RNA Signals**^b^			**CEP X Signals**^b^		
		**0**	**1**	**2**	**1**	**2**	**3**
			
81-58A (18)	euploid female	0.0 (0)	97.4 (442)	2.6 (12)	0.6^c^ (2)	99.4 (321)	0.0 (0)
82-6HT (22)	euploid male	100 (126)	0.0 (0)	0.0 (0)	99.0 (401)	1.0^c^ (4)	0.0 (0)
GM03623 (12)	trisomy X	0.0 (0)	6.7 (26)	93.3 (364)	nd^d^	nd	nd
							
GM04376 (11)	triploid XXX	0.0 (0)	99.7 (315)	0.3 (1)	0.0 (0)	4.8 (14)	95.2 (280)
GM10013 (7)	triploid XXX	0.0 (0)	90.0 (45)	10.0 (5)	0.0 (0)	3.5 (22)	96.5 (602)
GM10606 (8)	triploid XXX	0.0 (0)	97.3 (72)	2.7 (2)	nd	nd	nd
GM07744 (9)	triploid XXX	0.0 (0)	98.3 (113)	1.7 (2)	nd	nd	nd
							
GM04939 (7)	triploid XXX	0.0 (0)	51.2 (103)	48.8 (98)	0.0 (0)	10.8 (13)	89.2 (107)
GM04939 (10)	triploid XXX	0.0 (0)	51.8 (72	48.2 (67)	0.0 (0)	14.7 (26)	85.3 (151)
GM04939 (15)	triploid XXX	0.0 (0)	62.1 (100)	37.9 (61)	0.0 (0)	10.1 (26)	89.9 (222)
GM04939 (20)	triploid XXX	0.0 (0)	84.1 (90)	15.9 (17)	0.0 (0)	29.9 (59)	70.1 (138)
GM04939 (25)	triploid XXX	0.0 (0)	82.2 (106)	17.8 (23)	0.0 (0)	15.4 (39)	84.6 (214)
75-29 (6)	triploid XXX	0.0 (0)	27.7 (59)	72.3 (154)	0.0 (0)	4.0 (13)	96.0 (310)

^a^Passage numbers are in parentheses

^b^Percent of cells; cell numbers are in parentheses

^c^Most of these cells were quite large, and possibly tetraploid

^d^nd, not determined

### X enumeration

We analyzed cells for X chromosome number using a centromeric probe specific for X-linked alpha satellites (CEP X), as exemplified in Figure 1B. Expected patterns were found for male and female controls (Table 1), with over 99% of the female cells having two signals, and over 99% of the male cells having a single signal. The triploids all showed a significant X chromosome loss compared to normal female fibroblasts (p < 0.001 for GM04939; p < 0.01 for 75-29). GM04939 was especially striking in this respect, with about 10% of the cells having only two X chromosome signals.

### Cell selection

We noted that later passages of GM04939 (derived from a one-day-old neonate) had a reduced proportion of cells with two Xi's, and a study was set up to quantify this observation over time. GM04939 was followed for 10 passages, and the proportion of cells with two inactive X's dropped from 48% to 16% in one experiment, and from 38% to 18% in another (cell counts shown in Table 1). These changes are highly significant in a two-by-two chi square test (p < 0.001). In the same period, the proportion of cells with three X chromosomes dropped from 85% to 70% in the first experiment, and from 90% to 84% in the second (cell counts shown in Table 1). These changes in the numbers of X chromosomes are not statistically significant and they are considerably less than the decrease in the proportion of cells with two inactive X's. The loss of an X chromosome, therefore, could not be the major factor accounting for the decrease in proportion of XaXiXi cells with time in culture. As we show in the next section on "Clonal Analyses," selective overgrowth of XaXaXi cells appears to play a major role in the decrease in proportion of XaXiXi cells in culture.

### Clonal analyses

#### GM04939 triploid fibroblasts clones

We obtained 90 clones from GM04939, starting from a culture that consisted of approximately equal numbers of cells with one or two inactive X's (Table 2). Eighty-four of the clones consisted of cells with primarily or exclusively a single inactive X, while only six clones were detected that consisted primarily of cells with two inactive X's. We tried to score 100 cells or more per clone, which was always possible with the XaXaXi clones, but only occasionally possible with the XaXiXi clones, since these clones tended to be much smaller. This fact supports the observation of a selective growth advantage of XaXaXi cells over XaXiXi cells and suggests that the selective advantage may be due to a shorter generation time for the XaXaXi cells. It is likely that the selective advantage of XaXaXi over XaXiXi cells may be stronger under cloning conditions.

**Table 2:** XIST RNA variation in GM04939 clones^a^

**Clone Class**	**# Clones**	**XIST RNA Signals**^b^	
		**1**	**2**
		
Only XaXaXi	59	100 (6540)	0.0 (0)
Primarily XaXaXi	25	97.9 (3834)	2.1 (84)
Only XaXiXi	1	0.0 (0)	100 (32)
Primarily XaXiXi	5	7.7 (32)	92.3 (382)

^a^97% of cells have three X chromosomes

^b^Cell numbers are in parentheses

The 84 clones of the single inactive X class, XaXaXi, consisted of over 99% XaXaXi cells, with the majority (59) having only XaXaXi cells. Six clones, however, had over 2% XaXiXi cells, and two of these clones had 11% XaXiXi cells. There are several ways to explain the origin of these six mosaic clones. The clones could have originated from more than one cell, but because of the stringency with which we initiated these clones (see "Methods"), we feel that this possibility is extremely unlikely. Another possibility is that the cells with two signals are hexaploid, in which case the nuclei should be considerably enlarged; none of the cells with double signals appeared to be hexaploid, however, as we determined from nuclear size. Yet another possibility is that the doubles represent signals on sister chromatids, in which case the signals should be closely spaced together; we excluded such cases, however. The possibility we favor is that the clones originated from single XaXiXi cells, with an Xi being lost early in clonal growth, resulting in the predominance of cells with a single Xi in the expanded clone. We assume that after loss of an inactive X, duplication of an active X occurs so that the expanded clone is primarily XaXaXi. This assumption follows from the fact that our X enumeration analysis of GM04939 clones showed that average proportion of cells with three X's was over 96% (see the "*Paired XIST RNA and CEP X analyses*" subsection below).

#### 75-29 triploid clones

We obtained 37 clones from 75-29 (derived from a two-day-old neonate), starting with a culture in which 72% of the cells had two Xi's and 28% of the cells had one Xi. As shown in Table 3, twenty clones consisted exclusively of cells with single Xi's, and four clones had primarily cells with a single Xi. Seven clones consisted exclusively of cells with two Xi's, but four of these had very few cells to score (6 to 29). Six clones had primarily cells with two inactive X's. Because the clones originated from a culture with 72% of the cells having two inactive X's, the distribution of clones with respect to inactive X's is significantly different from expected (many fewer XaXiXi clones; χ^2 ^= 24.7, p < .001). As in the case of GM04939, the XaXiXi clones are smaller than the XaXaXi clones, supporting the conclusion that XaXaXi cells have a marked growth advantage over XaXiXi cells, which may be even stronger under cloning conditions.

**Table 3:** XIST RNA variation in 75-29 clones^a^

**Clone Class**	**# Clones**	**XIST RNA Signals**^b^	
		**1**	**2**
		
Only XaXaXi	20	100 (1955)	0.0 (0)
Primarily XaXaXi	4	94.6 (278)	5.4 (16)
Only XaXiXi	7	0.0 (0)	100 (371)
Primarily XaXiXi	6	3.8 (28)	96.2 (718)

^a^97% of cells have three X chromosomes

^b^Cell numbers are in parentheses

#### Paired XIST RNA and CEP X analyses

To see whether inactive X chromosome loss was involved in the formation of single-Xi cells, we carried out paired observations of XIST RNA and CEP X on 19 clones from GM04939 and 12 clones from 75-29. Each clone was split in two, and XIST RNA and CEP X assays were carried out individually on each half. If Xi loss is involved, one would expect to find clones of single inactive X cells that had only two X chromosomes. No such cells were found among 26 single-Xi clones. However, several single-Xi clones had one or more cells with two Xi's, and these clones had increased numbers of cells with only two X's. In one clone with several two-Xi cells, in particular, the frequency of cells with only two X's was greater than 15%. The mean frequency of cells with two X chromosomes in all the clones examined was less than 3%. We speculate that this unusual clone may have started from an XaXiXi cell and, during its early expansion, lost an inactive X and then duplicated an active X. A plausible explanation for a high frequency of XaXaXi versus XaXi triploid cells is that the two-Xa cells have a selective growth advantage over the one-Xa cells. In support of such events, loss of an inactive X followed by nondisjunction of an active X has been observed in cancer cells [10,11].

### Quantification of XIST RNA signals

**Figure 2:**
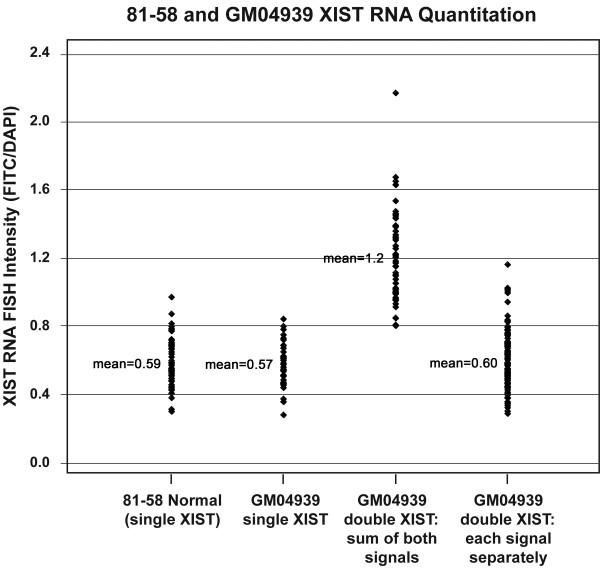
XIST RNA signal intensity distribution in control and triploid cells. Fluorescence intensities were quantified for XIST RNA FISH signals in normal diploid female fibroblasts (81-58), GM04939 XXX triploids with a single XIST signal, and GM04939 XXX triploids with two XIST signals (see "Methods"). For GM04939 cells with two XIST signals, the intensities of each signal are shown separately and in sum.

Although the XIST RNA bodies observed in the triploid cells appear normal in shape, we considered the possibility that the abnormal X:autosome ratio might affect the level of XIST accumulation on the inactive X. To obtain normalized XIST intensities in a variety of cells, we used epifluorescence imaging to quantify individual XIST FITC signals and compared it to their associated DAPI-stained signals. Such XIST:DNA ratios are shown in Figure 2 for GM04939 triploid fibroblasts and a normal female control. The distribution of intensities for individual signals do not vary appreciably between normal controls and GM04939 triploids, whether the latter have single or double signals. Summing the intensities in the GM04939 double-signal class results in a distribution of values that is approximately twice the single-signal distribution. All these data indicate that the levels of XIST RNA on the individual Xi's of triploid cells are normal despite the altered X:autosome ratio.

### DNA methylation

**Figure 3:**
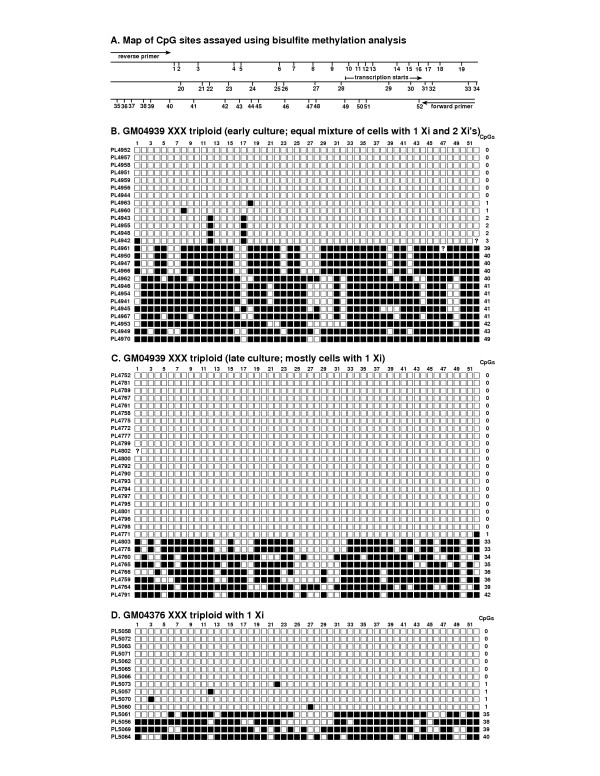
*G6PD *promoter methylation in control and triploid cells. A. Map of the 457 bp *G6PD *promoter region containing the CpG sites we analyzed for methylation (numbered 1–52). B. Methylation patterns from an early passage culture of GM04939 XXX triploid fibroblasts. CpG sites are represented as either methylated (■) or unmethylated (□); a question mark indicates that methylation status could not be determined. Each clone represents the methylation pattern of a single chromosome, and the number of methylated CpG's in each clone is given in the right column; clones are ordered from lowest to highest methylation. C. Methylation patterns from a late-passage culture of GM04939 XXX triploid fibroblasts. The proportion of hypermethylated clones (Xi alleles) is decreased in later passage cells, in agreement with the decrease in the number of Xi's seen cytologically. D. Methylation patterns from a culture of GM04376 XXX triploid fibroblasts that has a preponderance of cells with one Xi; the methylation patterns we observed are consistent with this deficit in Xi's relative to Xa's.

Because promoter methylation plays a major role in the maintenance of XCI, it was of interest to see if this was altered in triploid cultures. The promoter region of the X-linked *G6PD *gene (Fig. 3A) is known to be heavily methylated on the normal inactive X and hypomethylated on the active X [12]. We examined the methylation of this region in two mass cultures of GM04939 triploids and one mass culture of another XXX triploid, GM04376. One GM04939 culture analyzed was from an early passage that had two populations of cells in approximately equal proportion: one with single-XIST signals and one with double-XIST signals. The other culture studied was from a later passage that had a predominance of single-XIST signals. We cloned PCR amplimers from bisulfite-converted DNA to obtain methylation patterns along individual DNA strands; the region analyzed contains 52 potential sites of cytosine methylation (CpG's).

DNA's from normal female cultures result in hypomethylated and hypermethylated amplimer clones in approximately equal abundance ([12] and data not shown), as expected for the equal proportion of Xa (hypomethylated) and Xi (hypermethylated) alleles in normal female cells. An equal proportion of hypomethylated and hypermethylated alleles were also found in the early passage GM04939 culture, where the proportion of Xa's and Xi's in the cell population was approximately equal (Fig. 3B). As the proportion of active X's increased in later passage, the proportion of hypomethylated alleles also increased (Fig. 3C). Another XXX triploid culture with predominantly single-XIST signals, GM04376, also had an excess of hypomethylated versus hypermethylated alleles (Fig. 3D). Although our sampling of methylation patterns may not be extensive enough to yield strong statistical significance, the observed patterns are definitely consistent with normal methylation on the triploid inactive X, whether in cells with one or two Xi's.

## Conclusion

Our data suggest that the basic mechanisms of XCI appear to operate normally in triploids. Firstly, the XCI patterns are largely stable through cell division, as 70% of 127 clones had either all XaXaXi or all XaXiXi cells, and the remaining clones had over 95% of their cells sharing the same Xi pattern (either XaXaXi or XaXiXi). Second, XIST level and morphology on the Xi's in triploids, whether they have one or two, are comparable to those in normal female cells. Finally, DNA methylation of CpG island promoters on the inactive X, as evidenced by our *G6PD *analysis, appears similar to that of the inactive X in euploid female cells. Thus, both early (XIST RNA) and late (DNA methylation) features of the XCI process appear to be normal in triploids, even though the proper ratio of Xa's to autosomes cannot be achieved.

It is possible that the inviability of triploids results from the inability to achieve the ratio of one active X per diploid autosomal set. The inviability of tetraploids [13], however, argues against this idea because they can achieve the proper ratio. We speculate that our observation of X chromosome mitotic instability in triploids, as evidenced by an order of magnitude more X chromosome loss than seen in euploid loss (Table 1), may also apply to the autosomes. In addition, triploid/diploid mosaicism, which has been reported [14], implies abnormal segregation of whole chromosome sets in triploids. Perhaps mitotic chromosomal instability in triploids plays a major role in their inviability.

It is widely assumed that one of the earliest events in the initiation of XCI is a chromosome counting step that is required to achieve the one Xa per diploid autosomal set ratio. Studies in murine ES cells with deletions 3' to *Xist *show that the mutated X is preferentially inactivated even in XO and XY cells [15]. These results are interpreted by some workers [15,16] as indicating that these mutants are interfering with the chromosome counting process. An alternative interpretation is that the deletion mutants 3' to Xist could be interfering with a blocking site and not be related to chromosome counting. The earliest model for XCI proposed was that an episome, presumably independent of ploidy, would mark one X to be active by direct binding, with the remaining X or X's inactivated by default [17]. A later variant of this "counting to one" model proposed that the active-X marking factor would be autosomally derived and, therefore, dependent on ploidy [18-20].

The episome model would predict that at embryonic XCI, all cells in an XXX triploid would have one active X and two inactive X's, while the autosomal-dependent model would predict some degree of mosaicism. The murine data on triploids [8,9] report a very high proportion of cells with two inactive X's. We propose that human XXX triploids at embryonic XCI may also consist largely of cells with two Xi's, but that the selective advantage of XaXaXi cells over XaXiXi cells, plus possible losses of an inactive X, lead to the observed pattern of mainly XaXaXi cells. In the episome model for XCI, XaXaXi cells in triploids would have to originate by somatic segregation errors from parental XaXiXi cells, whereas under the autosomal-dependent marking model of XCI, XaXaXi cells would be normal products of the XCI process. This distinction permits a possible resolution between the two models.

If random XCI only forms XaXiXi cells at inception, and all the X's are distinguishable (e.g., X1, X2, X3), we would expect three classes of cells with respect to their patterns of active and inactive X's (X1 active, X2 and X3 inactive, etc.), in roughly equal abundance. If the XaXaXi cells are derived from XaXiXi cells by rare events such as Xi reactivation or segregational errors, they should exhibit a restricted pattern of allelic XCI, such as only X1 and X2 active, and X3 inactive. On the other hand, if the single-inactive X cells show varied XCI patterns, this would argue against the episome model of marking one X for activity and inactivating all remaining X's regardless of karyotype. Evaluation of these models will require studies of expressed polymorphic markers (ideally triallelic) in clonal triploid cultures.

In this study of dosage compensation in triploids, we have only considered XCI, the mechanism that brings about equivalence of X-linked gene expression between the sexes. Equally important in dosage compensation could be the transcriptional up-regulation of the single active X in each sex so as to avoid a haploinsufficiency effect. In *D. melanogaster*, transcriptional up-regulation of the male X has been known for some time [21], and the up-regulation complex, the "compensasome," that binds to the male X is reasonably well described [22]. In mammals, transcriptional up-regulation of the single active X in both males and females has recently been reported [23], but nothing is known about the underlying mechanism. For example, does the autosomal complement play a role in Xa up-regulation? Our triploid clones differing in XCI patterns should be of value in answering this question.

## Methods

### Triploids

We examined six human XXX triploid cultures: five from the NIGMS Human Genetic Cell Repository (GM04376, GM04939, GM07744, GM10013, and GM10606), and one (75-29) from Dr. George Martin's collection at the University of Washington (Table 1). GM04376, GM07744, GM10013, and GM10606 were derived from embryonic fetal specimens, while the GM04939 and 75-29 cultures were derived from one-day-, and two-day-old newborns, respectively. All cultures were previously characterized cytogenetically; we confirmed triploidy in GM04939, and carried out cytological X chromosome enumeration studies on GM10013, GM04376, GM04939, and 75-29 using an alpha satellite probe (Vysis, Downers Grove, IL).

### Cell culture

The cells were grown in AmnioMax™-C100 medium (Invitrogen Gibco, Grand Island, NY) and harvested in trypsin/EDTA (Invitrogen Gibco). Cloning was carried out by aliquoting 1 μl of a dilute cell suspension from an early passage into wells of a 96-well flat-bottomed tissue culture plate and visually scoring wells for single cells. These wells were followed for growth and approximately 10 percent were positive for clones. Clones were then transferred to wells of a 24-well tissue culture plate for limited expansion and then used for FISH analysis by plating trypsinized cells onto coverslips in 35 mm petri dishes (MatTek, Ashland, MA).

### FISH assays

#### XIST RNA

Cells grown on coverslips were fixed in 1% formaldehyde (Ted Pella, Redding, CA) plus 0.5% Triton X-100 for 10 min at room temperature. Cells were dehydrated in ethanol washes, air-dried, and used immediately for hybridization. The XIST probe, pXIST GIA (a gift from C. Brown, Univ. of British Columbia), was labeled either by DIG-nick translation (Roche Diagnostics, Indianapolis, IN) or by the SpectrumGreen™ direct label kit from Vysis (Downers Grove, IL). Hybridization and detection were carried out according to the manufacturers' instructions. Over 95% of the cells could be scored for XIST RNA signals. Those that could not be scored, which may have included some truly XIST RNA-negative cells, were necessarily excluded from our analyses.

#### X chromosome enumeration

Cells grown on coverslips in 35 mm petri dishes were fixed in 3:1 methanol:acetic acid, air-dried, placed for 1 hr in 2X SSC at 37°C, dehydrated in ethanol washes, and air-dried. Hybridization with the CEP X (DXZ1) alpha satellite probe (Vysis, Downers Grove, IL), which was used for counting X chromosomes in interphase cells, was carried out according to the manufacturer's instructions.

#### Genomic DNA isolation and methylation analysis

Genomic DNA from fibroblasts was purified by the Puregene^® ^purification kit (Gentra, Minneapolis, MN) according to the manufacturer's instructions. The bisulfite modification method [24] was used to analyze DNA methylation profiles at the *G6PD *locus following the detailed procedures of Hansen et al. [12]. Briefly, DNA's were treated with sodium bisulfite and used as a template for PCR amplification using primers specific to the converted bottom strand of the *G6PD *promoter region (Fig. 3A), and the products were cloned and sequenced using standard protocols. Sequence traces were assembled and manually verified using Sequencher software version 4.2 (Gene Codes, Ann Arbor, MI).

#### Quantification of XIST RNA signals

Quantification of fluorescent XIST RNA signals was carried out by epifluorescence imaging with a Marianas imaging system consisting of a CoolSnap HQ camera, ZeissAxiovert 200 M inverted microscope, and a liquid light guide-coupled 175 W xenon lamp, all controlled by Slidebook software (Intelligent Imaging Innovations, Santa Monica, CA). Images were collected using a 40X PlanNeoFluor objective. Threshold limits were set on captured images to isolate the DAPI and FITC signals, and the Slidebook software was used to quantify these signals.

## Abbreviations

XCI, X chromosome inactivation

Xi, inactive X chromosome

Xa, active X chromosome

## Authors' contributions

SG and RSH conceived the study design, supervised and coordinated its progress, and drafted and prepared the final manuscript. SG, TN, KV, and PL carried out the cell culture and cytological studies. TC, PL, and RSH carried out the bisulfite methylation analyses. SG carried out statistical analyses. All authors read and approved the manuscript.

**Table 4:** X chromosome number in clones that are pure or mosaic for XIST RNA signals

**Clone Type**	**X Signals (# cells)**		**Percentage of**
	**2**	**3**	**Cells with 2 X's**
		
One inactive X (22 clones)	67	3584	1.8
Both 1 and 2 inactive X's (4 clones)	67	824	7.5*

* p < 0.001
